# Optimization of saccharification potential of recombinant xylanase from *Bacillus licheniformis*

**DOI:** 10.1080/21655979.2017.1373918

**Published:** 2017-09-28

**Authors:** Muhammad N. Aftab, Asma Zafar, Irfana Iqbal, Afshan Kaleem, Khalid M. Zia, Ali R. Awan

**Affiliations:** a Institute of Industrial Biotechnology, Government College University, Katchery Road, Lahore, Pakistan; b Department of Zoology, Lahore College for Women University, Lahore, Pakistan; c Department of Biotechnology, Lahore College for Women University, Lahore, Pakistan; d Institute of Chemistry, Government College University, Faisalabad, Pakistan; e Institute of Biochemistry & Biotechnology, University of Veterinary and Animal Sciences, Lahore, Pakistan

**Keywords:** Xylanase, expression, biomass, saccharification

## Abstract

Saccharification potential of xylanase enzyme cloned from *Bacillus licheniformis* into *E. coli* BL21 (DE3) was evaluated against plant biomass for the production of bioethanol. The expression of cloned gene was studied and conditions were optimized for its large scale production. The parameters effecting enzyme production were examined in a fermenter. Recombinant xylanase has the ability to breakdown birchwood xylan to release xylose as well as the potential to treat plant biomass, such as wheat straw, rice straw, and sugarcane bagass. The saccharification ability of this enzyme was optimized by studying various parameters. The maximum saccharification percentage (84%) was achieved when 20 units of recombinant xylanase were used with 8% sugarcane bagass at 50°C and 120 rpm after 6 hours of incubation. The results indicated that the bioconversion of natural biomass by recombinant xylanase into simple sugars can be used for biofuel (bioethanol) production. This process can replace the use of fossil fuels, and the use of bioethanol can significantly reduce the emission of toxic gases. Future directions regarding pre-treatment of cellulosic and hemicellulosic biomass and other processes that can reduce the cost and enhance the yield of biofuels are briefly discussed.

Xylanase is one of the important hemicellulases which hydrolyses the xylan backbone to release short xylooligomers and xylose from hemicelluloses. The short oligomers can be further broken down into single units of xylose by β-xylosidase.^1,2^ Both endo-1,4-β-xylanase and β- xylosidase enables the production of xylose as a major industrial raw material. The activity of endo-1,4-β-xylanase produces non-reducing ends of xylooligosaccharides from which xylose is released by enzymatic action of β-xylosidases.^3^ In this addendum, the optimization of the various parameters involved in the enhanced expression of recombinant xylanase enzyme as well as for the maximum saccharification of plant biomasses are discussed.

The cell walls of plants are the chief repository of fixed carbon in the environment, and are comprised of three main polymeric components: hemicellulose (non-cellulosic polysaccharides), cellulose (insoluble fibers of 3–1,4-glucan) and lignins (with a complex polyphenolic structure).^4^ After cellulose, xylan is the second most abundant polysaccharide on earth that constitutes one-third of surrounding renewable organic carbon.^5^ The plant cell wall polysaccharides produce easily accessible sugar residues that can be utilized in biotechnological and industrial processes.^6^ The conversion of xylan into valuable products represents a significant portion of the efforts to attain economical feasibility of the lignocellulose biomass processing and to conflict with chemicals and renewable vitality as well.^7^

A coordinated series of biochemical processes result in the degradation of plant cell wall components. The continuous action of enzymes lead to a complete hydrolysis of the hemicelluloses into monosaccharides.^8^ The microbial breakdown of lignocellulosic biomass is gaining importance for the production of renewable biofuels to address global energy requirements.^9^ Many types of enzymes are involved in the complete hydrolysis of xylan into its constituent sugars for biofuel fermentation. For hydrolyzing xylan backbone into xylooligosaccharides and individual xylose units, two enzymes, endo-β- xylanase and β-xylosidase, are extremely important^2^ in which xylanase (1,4-β-D-xylan xylanohydrolase) plays a crucial role in cleavage of core linkages on the β-1,4-xylose backbone.^10^

For the large-scale production and commercial feasibility of any industrially important enzyme, an economical substrate and 30–40% proficient fermentation process are crucial.^11^ Advancements in genetic engineering techniques facilitate the enhanced production of different industrially important enzymes.^12^ Significant improvements in these techniques resolved many problems, such as insufficient enzyme stability, operational stability and reactivity with a wide range of substrates.^13^

In the present study, the saccharification ability of the cloned xylanase enzyme from *B. licheniformis*^14^ on plant biomass has been evaluated. In addition, the expressions of the cloned xylanase gene has been optimized by fermenter studies. The xylanase gene of *B. licheniformis* was cloned and successfully expressed into *E. coli* by using pET-22b(+) expression vector (Fig. 1). The expression of recombinant xylanase was analyzed by SDS-PAGE (Fig. 2) by following the method of Laemmeli.^15^ Different controls, including intracellular fractions of wild *E. coli* BL21 (DE3), non-induced *E. coli* containing only the expression vector without any insert, induced *E. coli* containing only expression vector without any insert and non-induced *E. coli* containing expression vector with insert were also run in parallel. A distinct band at 23 kDa (Fig. 2, lane 6) indicated the successful expression of xylanase gene. Recently, a xylanase gene from *Bacillus subtilis* into *E. coli* by using pET-22b(+) vector was expressed (21 kDa enzyme)^16^ and the expression of 23.3 kDa xylanase enzyme after cloning of *Bacillus tequilensis* xylanase gene into *E. coli* by using pCR® 2.1-TOPO® TA cloning vector was reported.^17^

**Figure 1.**
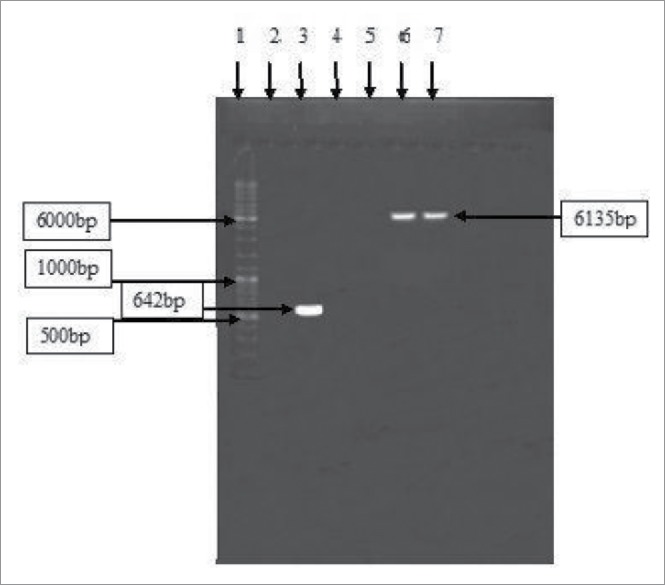
Amplified gene of xylanase from *Bacillus licheniformis* on agarose gel. GeneRuler™ DNA marker is present in lane 1 while in lane 3 amplified xylanase gene (642 bp) can be seen in lane 1 while lanes 6 and 7 show recombinant pET 22b(+) containing xylanase gene (6135 bp).

**Figure 2.**
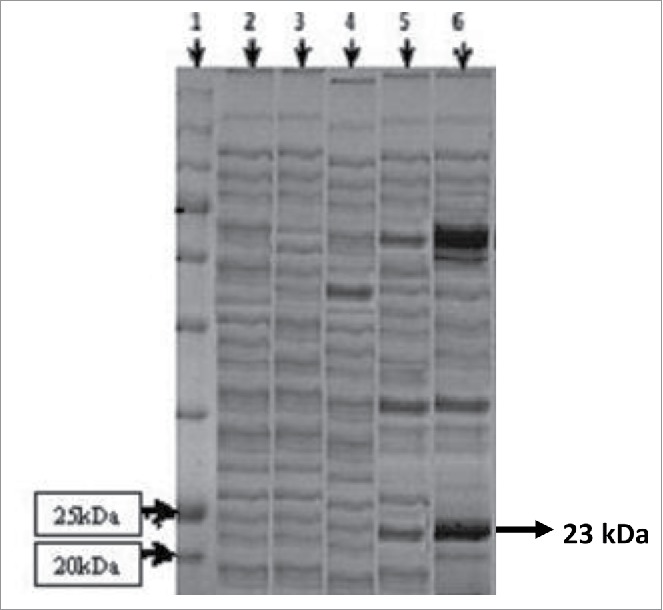
SDS-PAGE analysis for expression of of cloned xylanase gene. Protein ladder, cell lysate of wild *E. coli* BL21 (DE3), non-induced vector [pET-22b (+)] only, induced vector [(pET-22b(+)] only, no-induced recombinant vector contains xylanase gene and induced recombinant vector containing xylanase gene are present in lanes 1, 2, 3, 4, 5 and 6, respectively. Distinct band of 23 kDa in lane 6 shows the successful expression of recombinant xylanase.

Expression of recombinant xylanase enzyme was optimized in a 7.5 L glass fermenter fixed with a round bottom flask of 5 L. Different parameters, such as the agitation rate (100 – 300 rpm), aeration rate (0.5 – 3.0 vvm), inoculum size (1 – 6%), dissolved oxygen concentration (10 – 30%) and media volume (40 – 90%) were optimized as shown in Fig. 3 to achieve maximum expression of the cloned xylanase gene. It was observed that the maximum expression was obtained when aeration rate was 2.0 vvm, agitation rate was 200 rpm, dissolved oxygen level was 20%, volume of medium was 70% of the total volume and size of inoculum was 2% (Fig. 3). The maximum production of recombinant xylanase from *Bacillus subtilis* was reported in a 6 L fermenter at 0.6 vvm aeration rate and 150 rpm agitation speed.^18^ For maximum xylanase production from *Aureobasidium pullulans* 200 rpm agitation rate and 1.5 vvm aeration rate has been reported.^19^ In another study, the maximum xylanase enzyme production was achieved from *Bacillus altitudinis* in a 7.5 L stirred tank fermenter with 12.5% inoculum size when the agitation and aeration rates were 200 rpm and 1.00 vvm, respectively.^20^

**Figure 3.**
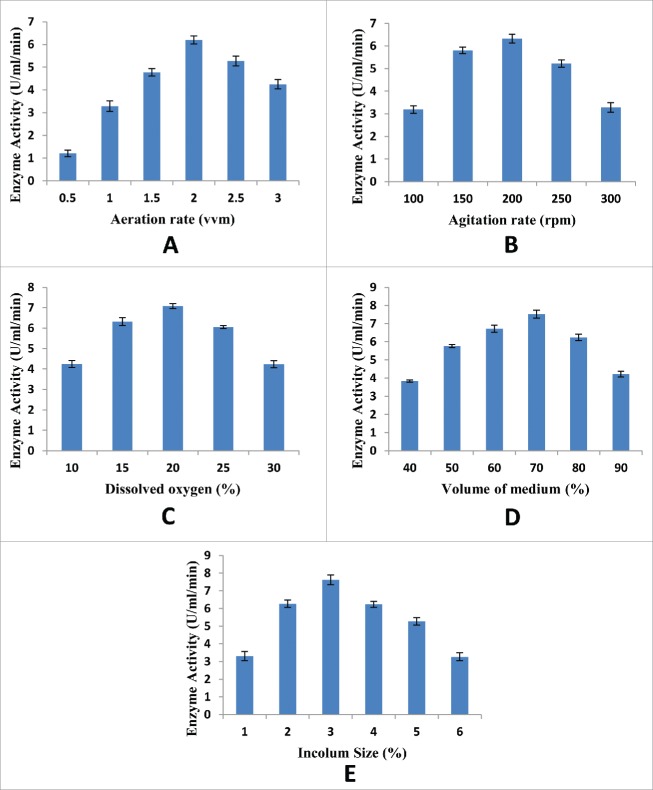
Optimization of enzyme expression of recombinant xylanase in 7.5 L glass fermenter. (A) Effect of aeration rate (B) Effect of agitation rate (C) Effect of dissolved oxygen (D) Effect of volume of medium (E) Effect of inoculum size. Y-error bars show the standard deviation (S.D) of parallel replicates (n=3). Each mean value differ significantly at p ≤ 0.05%.

The saccharification potential of the recombinant xylanase enzyme was tested for various pre-treated plant biomasses including wheat straw, rice straw and sugarcane bagass as shown in Fig. 4. Different parameters including temperature (30–70°C), incubation time (2- 24 hours), buffer pH (5–9), enzyme concentration (5–70 units), substrate concentration (1–10%) and agitation speed (60–180 rpm) were studied in order to determine the maximum percentage of saccharification. The results revealed that almost 84% saccharification was achieved when recombinant xylanase enzyme (20 units) was incubated with 8% pre-treated sugarcane bagass at 50°C in a pH 6.0 buffer after 6 hours of incubation. It was also reported that a xylanase enzyme from *Bacillus pumilus* which was capable of hydrolyzing wheat straw gave maximum saccharification results after 6 hours of incubation at 40°C when 500 units of enzyme were used.^21^ These results are comparable with those of Dhabhai et al. who had reported the maximum saccharification from Pearl millet straw in comparison with wheat straw and sugarcane bagass at 50°C in a buffer of pH 5.0.^22^ In another study, it was reported that bagass is the best natural substrate for the xylanase enzyme from *Bacillus subtilis* which produced maximum xylanase activity as compared to wheat straw, rice straw and wheat bran.^23^

**Figure 4.**
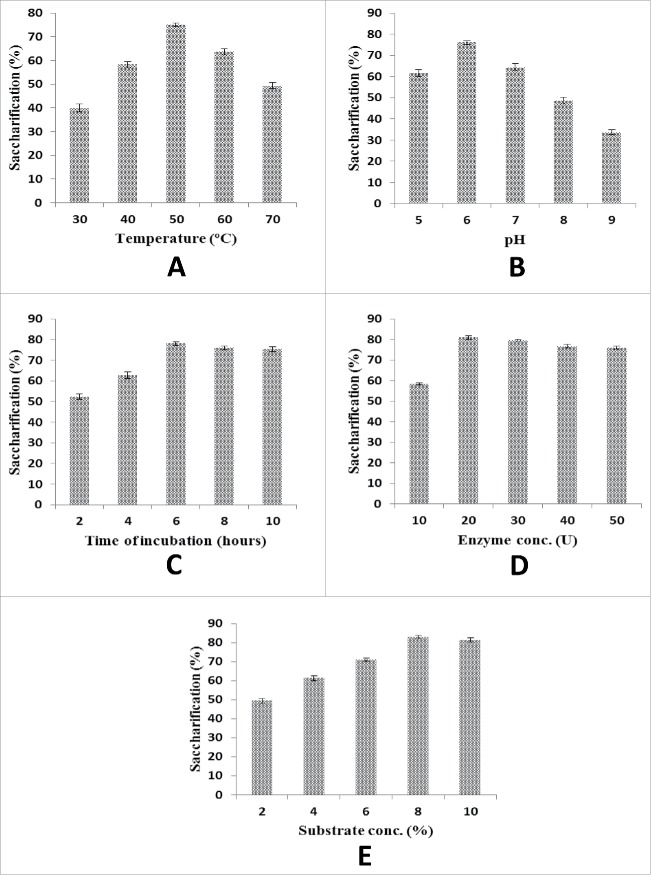
Optimization of saccharification potential of recombinant xylanase enzyme (A) Effect of incubation temperature (B) Effect of pH (C) Effect of time of incubation (D) Effect of enzyme concentration (E) Effect of substrate concentration. Y-error bars show the standard deviation (S.D) of parallel replicates (n=3). Each mean value differ significantly at p ≤ 0.05%.

The economic status and growth rate of developing countries all over the world are related to their utilization of energy sources.^24^ As fossil energy sources are gradually depleting due to the increase in the world's population and its use in vehicles and by industry, the shortage of fossil energy sources has remained a major global topic for debate. On the other hand, the problem of environmental pollution related to fossil fuels due to the emission of greenhouse gases is another major global problem.^25^ To overcome the dilemma of the depletion of fossil energy reserves and to combat the increasing demand of energy and the reduction of emissions, a series of new concepts and strategies have been developed for the production of alternative energy sources which are environmentally friendly, economical and bio-degradable.^26^ The production of liquid biofuel from renewable resources provides an attractive solution to the problems of excessive reliance on fossil energy fuels alongside environmental concerns and also provides energy security to the world.^27^ Bioethanol is the most dominant renewable energy source in the world, and emerges as a promising transport biofuel for transport and depending on the feedstock, offers up to 80% saving of the harmful emissions over conventional fossil fuels.^28^ Only 3.0% of the world's total energy requirements is being fulfilled by the renewable sources but by 2050 it could potentially increase to 20 – 80% of the total global energy consumption.^29^

A promising renewable resource for bioethanol production is lignocellulosic biomass, which is also used as a raw material for the production of other chamicals as well.^30^ Biomass can potentially be utilized without the risk of depletion due to its renewable nature; however, it poses numerous challenges to the technologies used for its conversion due to its structural features. An efficient conversion strategy must therefore be adopted for the effective processing of lignocellulosic biomass that guarantees exploitation of every part of the biomass.^26^ Most ethanol production processes involve the pretreatment of the lignocellulosic biomass, followed by enzymatic hydrolysis in order to produce fermentable sugar that can subsequently be converted into ethanol by the application of a suitable yeast strain.^31,32^ Process economics is strongly influenced by both steps, i.e., pretreatment and saccharification.^33^ Up till now, different pretreatment technologies have been developed which can be categorized as physical, biological, physicochemical, chemical or a combination of all of these.^34^ Amongst these, the steam explosion method is considered to be one of the most efficient pretreatment methods, given that this seem more environmentally friendly and cost-effective on an industrial scale.^35^ The biological pretreatment method, in which various metabolites from microorganisms are used, is also considered to be environmentally friendly because no chemicals are used in this method and it does not involve recycling because the by-products produced do not interfere in saccharification process.

Pretreated lignocellulosic biomass is converted into fermentable sugars by the action of various cellulolytic and xylanolytic enzymes as well as accessory enzymes. the complete hydrolysis of the hemicellulose contents of biomass requires two types of enzymes namely xylanases and xylosidases, while enzymes that degrade cellulose contents fall into three types namely, endoglucanases, exoglucanases and glucosidases. Accessory enzymes, like pectinases, arabinases, lyases, estrases and glactanases, are required in minute quantities alongside these cellulases and hemicellulases. Theses enzymes are being used individually or together for the efficient liberation of sugar residues from pretreated biomass which are further fermented into ethanol. An effective strategy for enzymatic saccharification of pretreated biomass provides good yield of fermentable sugars which in turn results in high yield of bioethanol by application of fermentation technology. The major limitations in enzymatic saccharification of plant biomass are the hydrolytic efficiency and cost of the process. To overcome these problems, several studies have been conducted to improve the process viability and to make it cost-effective. There is a need to establish more proficient and cost-effective methods to produce effective enzymes and also to study their kinetics in order to design the efficient production for bioethanol. For example, enzymes can be reused by immobilization on a solid support in order to reduce the processing cost.

The fermentation of pretreated lignocellulosic biomass is being carried out by the application of various microorganisms including bacteria, fungi and yeast. The most commonly used organism for ethanol production is yeast, *Saccharomyces cerevisiae*, however, some bacteria, such as *Zymomonas mobilis* and *Pichia stipites* are also used in ethanol fermentation. The major rate- limiting factor in ethanol fermentation is the incomplete utilization of all the sugars products including pentoses and hexoses by these naturally occurring microorganisms. Much effort has been employed to overcome this problem by genetically modifying several microorganisms to permit the fermentation of both pentoses and hexoses at a comparatively high rate. The purification of bioethanol is also a critical step before use of enzyme in saccaharification process. The fermented product is generally separated into ethanol and water by the process of distillation and upto 95% ethanol can be obtained. Nevertheless, distillation has many disadvantages including cost of processing. However, many other ethanol purification techniques are being employed, such as adsorption, ozonation and gas stripping. The purity of the ethanol is checked by various techniques, including gas chromatography, high performance liquid chromatography, infra-red spectroscopy and olfactometry.
